# Supplementary material for: Interactions between Ambient Air Particles and Greenness on Cause-specific Mortality in Seven Korean Metropolitan Cities, 2008–2016
Source: Int J Environ Res Public Health. 2019 May 27;16(10):1866. doi: 10.3390/ijerph16101866 (PMC6572360; doi:10.3390/ijerph16101866)
Supplement: Supplementary file 1 [file ijerph-16-01866-s001.pdf]

Supplementary Materials

Interactions between Ambient Air Particles and Greenness on Cause-specific Mortality in Seven Korean Metropolitan Cities, 2008–2016

Sera Kim [1], Honghyok Kim [1] and Jong-Tae Lee [1,2,*]

[1] BK21PLUS Program in 'Embodiment: Health-Society Interaction', Department of Public Health Science, Graduate School, Korea University, Seoul, 02841, Korea; ssera0905@gmail.com (S.K.); honghyok@korea.ac.kr (H.K.)

[2] School of Health Policy and Management, College of Health Science, Korea University, Seoul, 02841, Korea

* Correspondence: jtlee@korea.ac.kr; Tel.: +82-2-3290-5668

Table of contents

1. Description of study variables

Figure S1. The location of study area ··· 3

Figure S2-9. Spatial distribution of study variables ··· 4

Figure S10-17. Annual trend of study variables ··· 8

2. Results of sensitivity analyses

Table S1. Spearman correlation coefficients ··· 9

Table S2. Percent changes in cause-specific mortality and 95 % confidence interval for 10 μg/m^3 increase in PM_{10} in two-pollutant model ··· 9

Table S3. Percent changes in cause-specific mortality and 95 % confidence interval for 10 μg/m^3 increase in PM_{10} using models with different socioeconomic status indicators ··· 10

Table S4. Percent changes in cause-specific mortality per 10 μg/m^3 increase in PM_{10} by the level of greenness using models with different socioeconomic status indicators ··· 11

Table S5. Percent changes in cause-specific mortality and 95 % confidence interval for IQR increase in NO_2 and SO_2 in single- and two-pollutant model ··· 12

Figure S18. City-specific percent changes in cause-specific mortality and 95 % confidence interval for 10 μg/m^3 increase in PM_{10} ··· 13

Figure S19. City-specific percent changes in cause-specific mortality and 95 % confidence interval for IQR increase in NDVI ··· 14

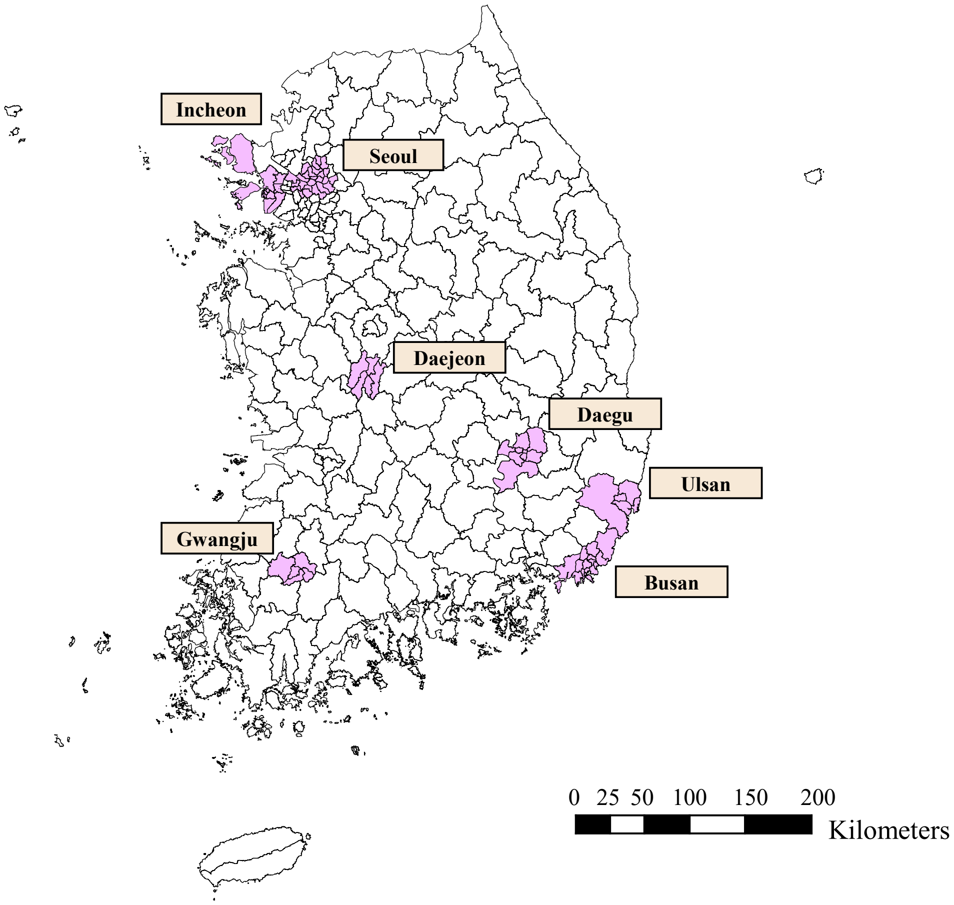

Figure S1. The location of study area (seven metropolitan cities in South Korea).

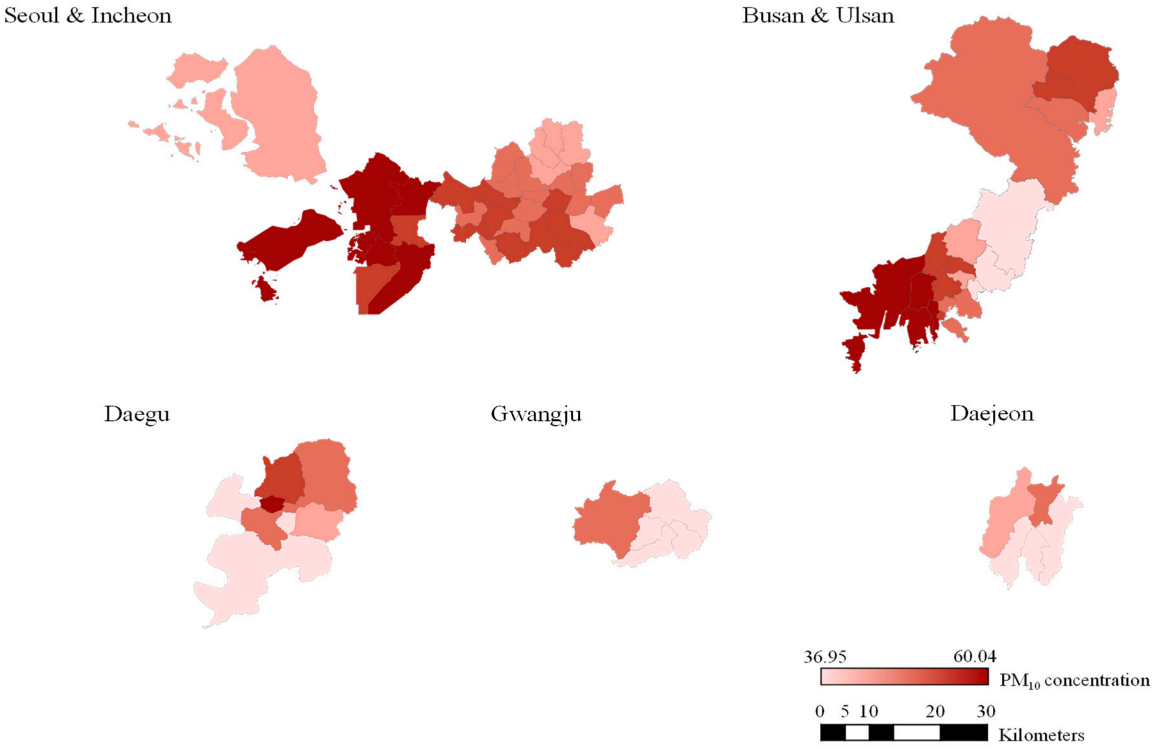

Figure S2. Distribution of PM_{10} concentrations in µg/m^3 (averages for 2008–2016).

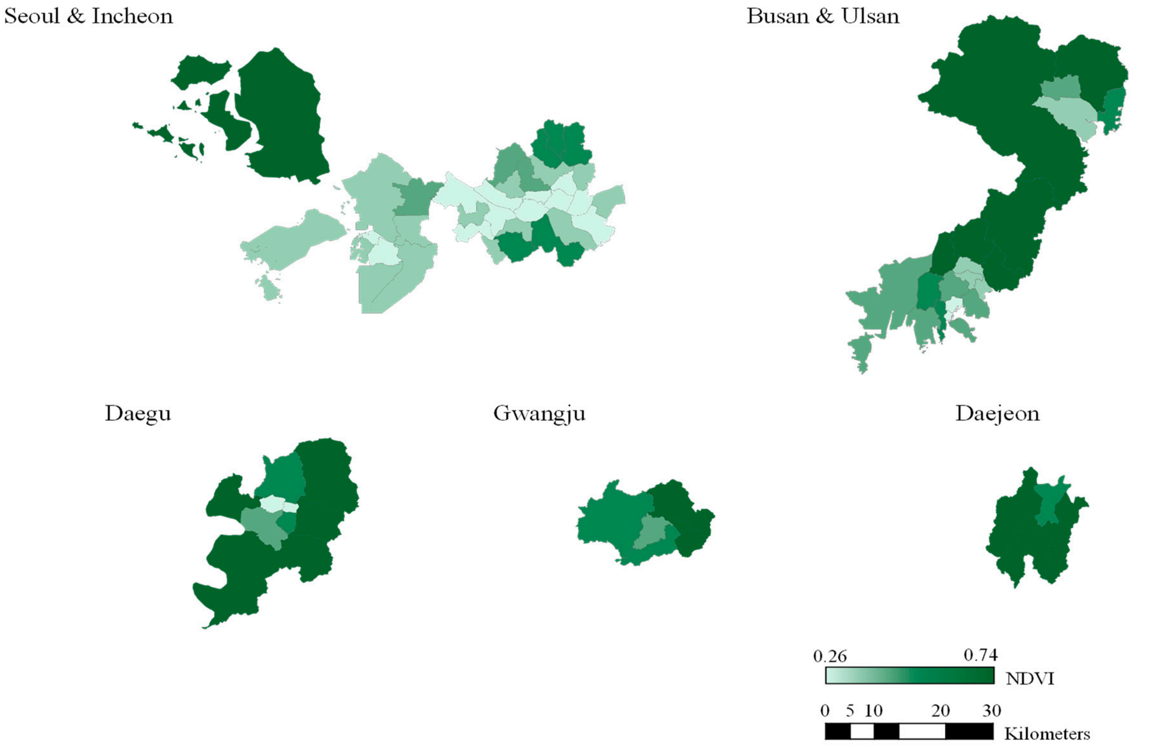

Figure S3. Distribution of greenness by NDVI (averages for 2008–2016).

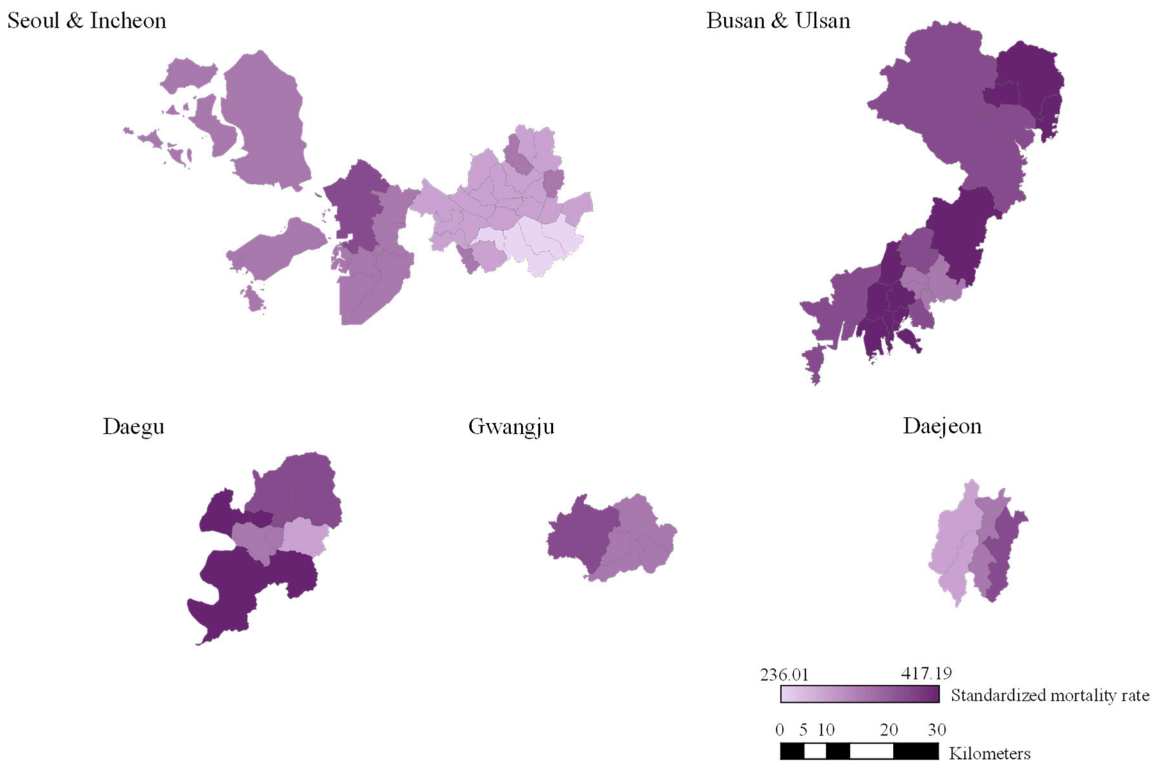

Figure S4. Distribution of standardized non-accidental mortality rates. (per 100,000 / averages for 2008–2016).

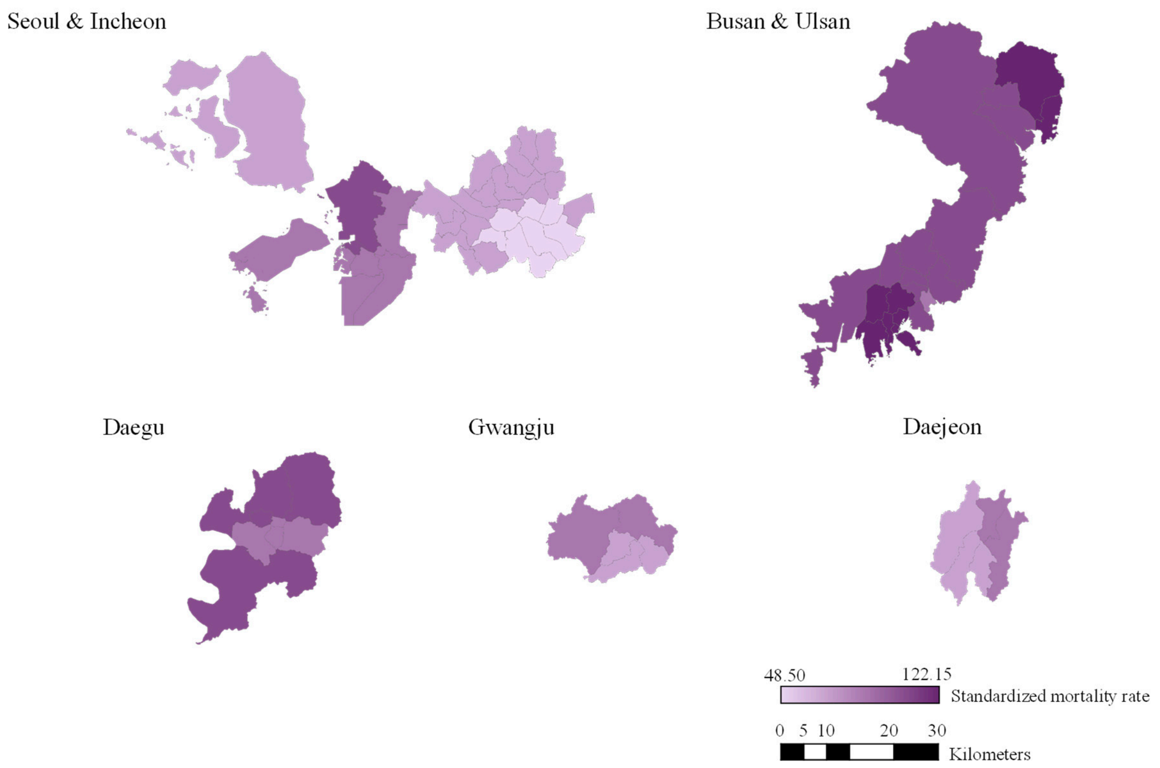

Figure S5. Distribution of standardized cardiovascular mortality rates. (per 100,000 / averages for 2008–2016).

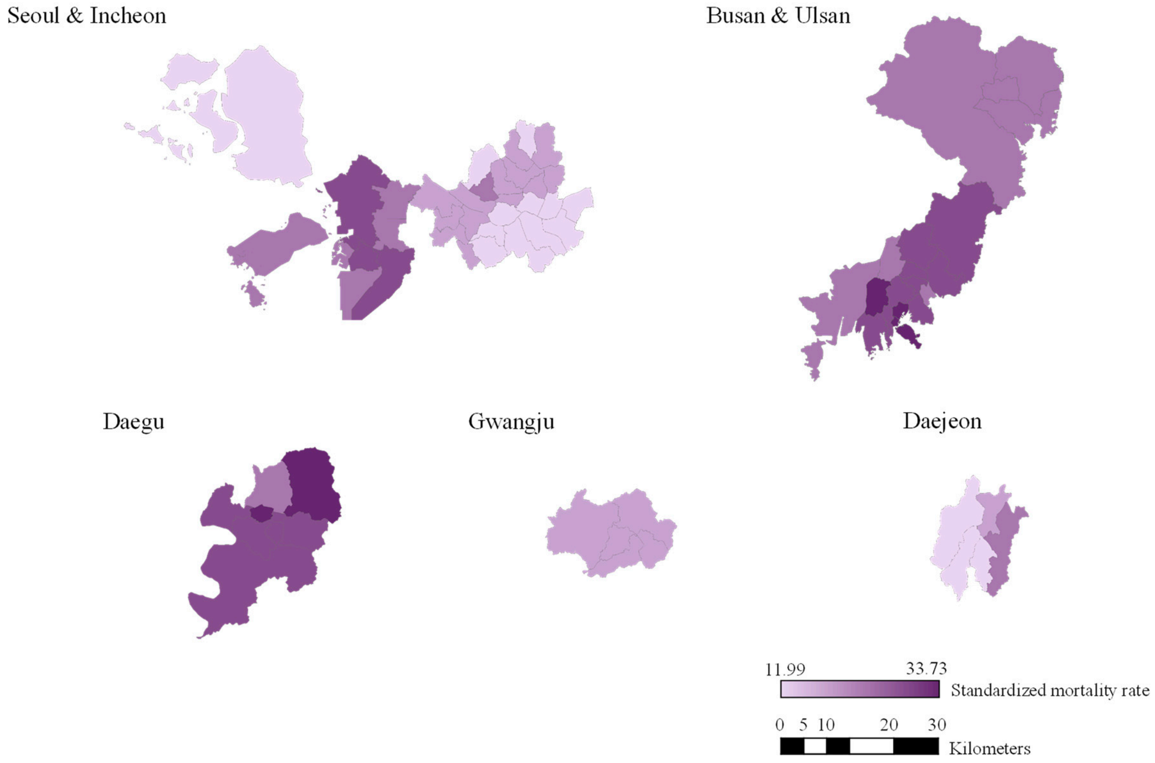

Figure S6. Distribution of standardized ischaemic heart disease mortality rates (per 100,000 / averages for 2008–2016).

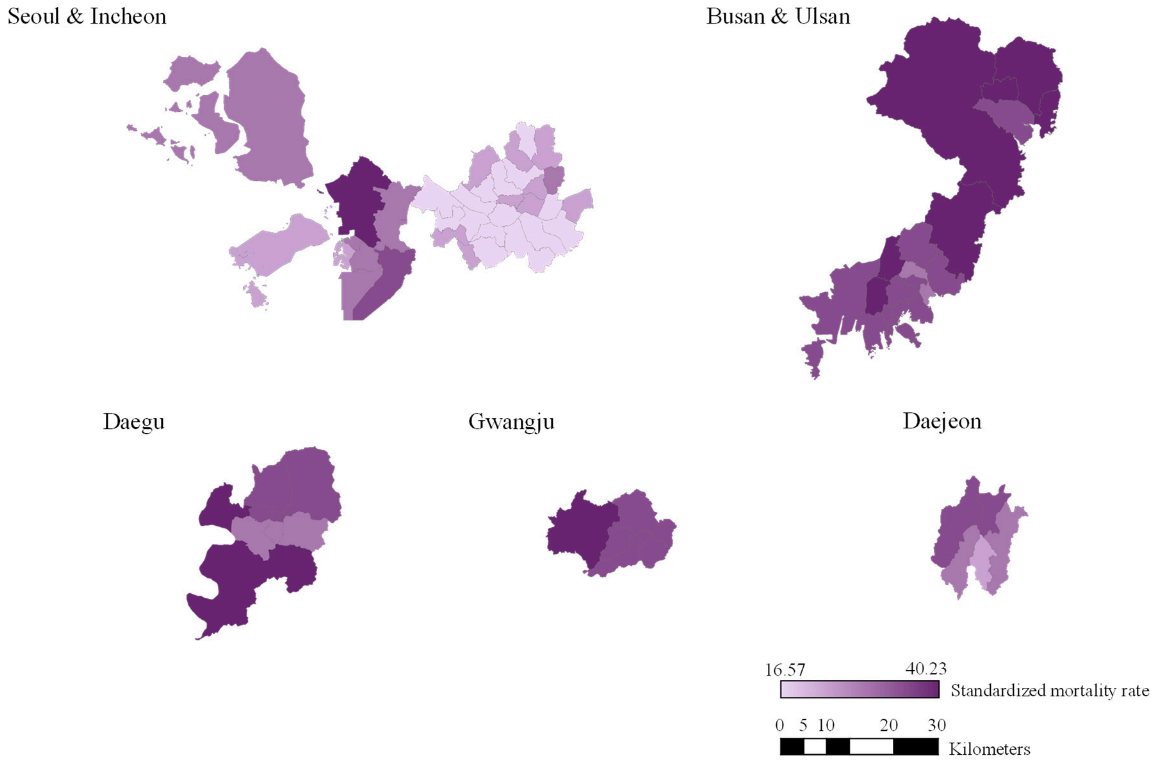

Figure S7. Distribution of standardized respiratory disease mortality rates (per 100,000 / averages for 2008–2016).

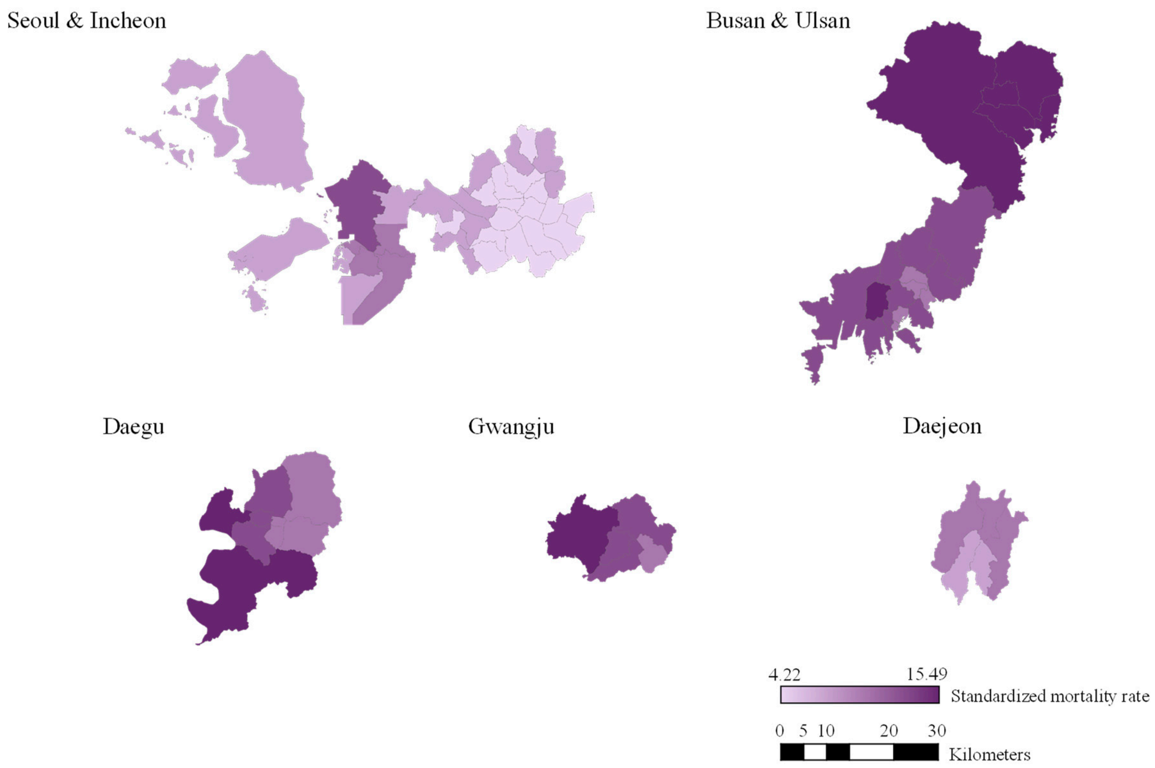

Figure S8. Distribution of standardized chronic lower respiratory mortality rates. (per 100,000 / averages for 2008–2016).

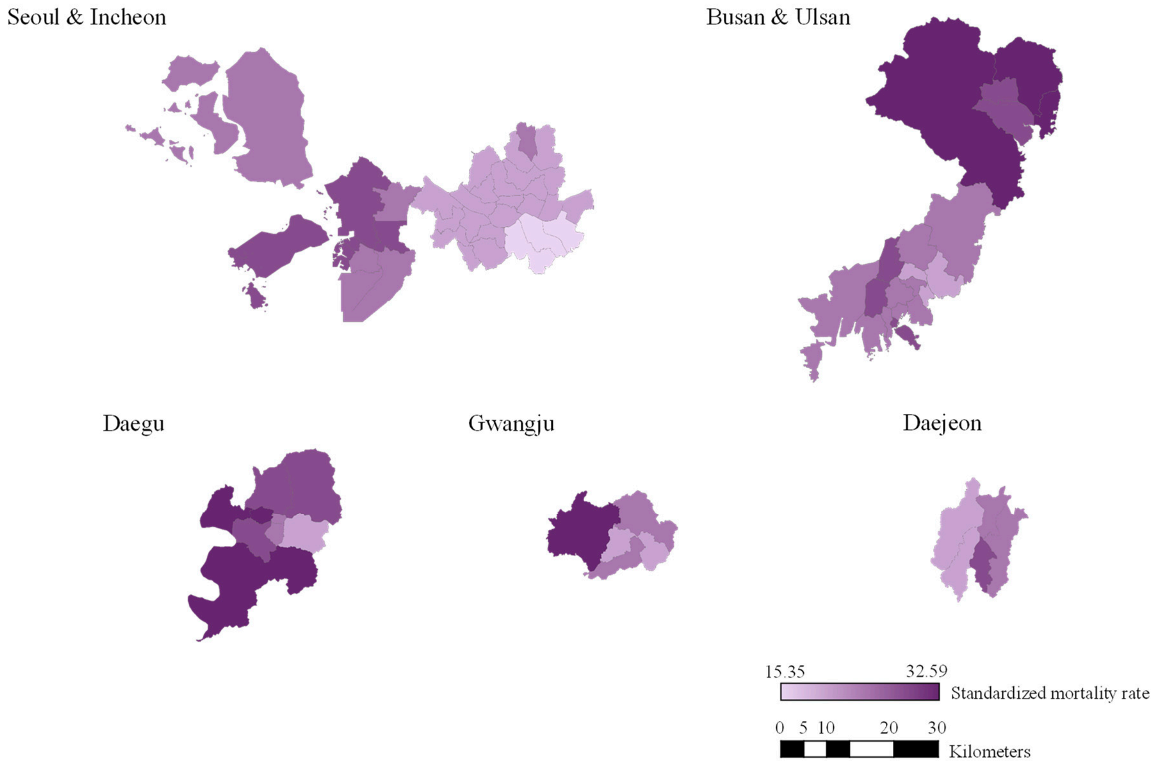

Figure S9. Distribution of standardized lung cancer mortality rates (per 100,000 / averages for 2008–2016).

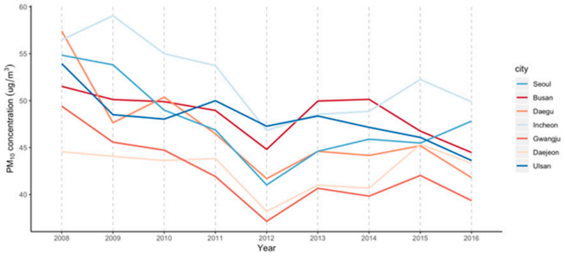

Figure S10. Annual trend of PM_{10} concentration.

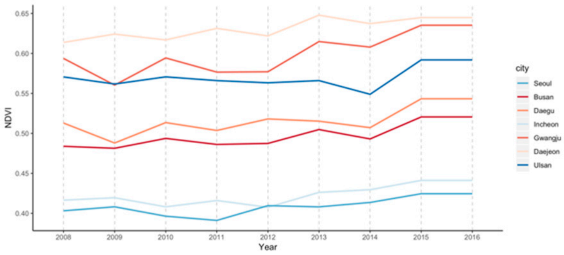

Figure S11. Annual trend of NDVI.

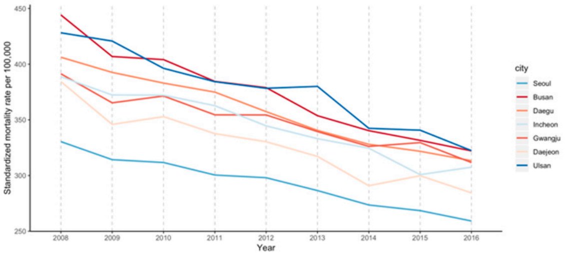

Figure S12. Annual trend of standardized non-accidental mortality rates.

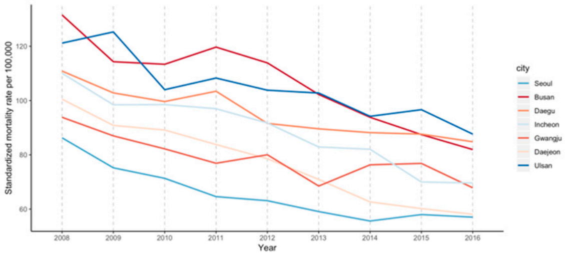

Figure S13. Annual trend of standardized cardiovascular mortality rates.

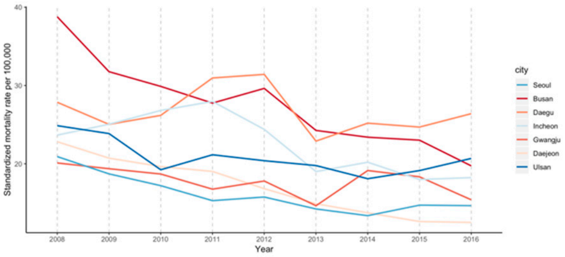

Figure S14. Annual trend of standardized ischaemic heart disease mortality rates.

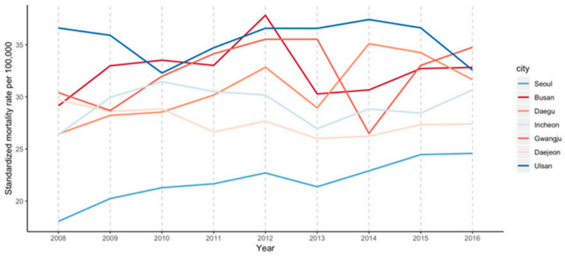

Figure S15. Annual trend of standardized respiratory mortality rates.

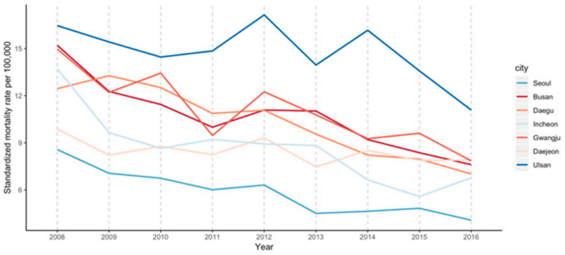

Figure S16. Annual trend of standardized chronic lower respiratory disease mortality rates.

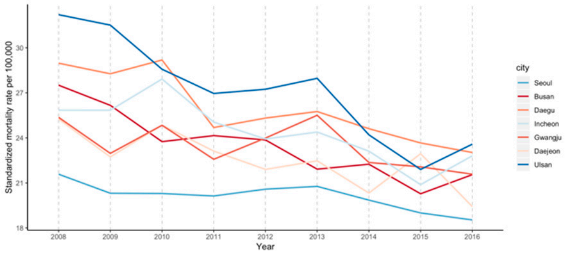

Figure S17. Annual trend of standardized lung cancer mortality rates.

Table 1. Spearman correlation coefficients.

	PM_{10}	NDVI	Percentage of Adults with Low Education	Smoking Rate	Health Care Resource Index
PM_{10}		-0.29*	0.16*	0.29*	-0.11*
NDVI			0.18*	-0.10*	-0.04
Percentage of adults with low education				0.58*	0.17*
Smoking rate					0.00
Health care resource index					

* p-value < 0.05

Table S2. Percent changes in cause-specific mortality and 95 % confidence interval for 10 μg/m³ increase in PM_{10} in two-pollutant model.

	Non-Accidental	Cardio Vascular Disease	Ischaemic Heart Disease	Respiratory Disease	Chronic Lower Respiratory Disease	Lung Cancer
Single pollutant (PM_{10})	4.49 (3.41, 5.57)	9.70 (7.64, 11.81)	7.50 (4.19, 10.90)	-3.12 (-5.36, -0.83)	16.03 (11.42, 20.85)	2.98 (0.92, 5.08)
+ SO_2	4.59 (3.51, 5.69)	9.82 (7.72, 11.96)	7.32 (3.96, 10.79)	-3.23 (-5.50, -0.91)	16.42 (11.76, 21.28)	3.29 (1.19, 5.44)
+ NO_2	4.18 (3.08, 5.30)	8.93 (6.83, 11.08)	6.89 (3.49, 10.40)	-2.36 (-4.72, 0.05)	14.98 (10.24, 19.93)	2.91 (0.78, 5.09)

Table S3. Percent changes in cause-specific mortality and 95 % confidence interval for 10 μg/m³ increase in PM_{10} using models with different socioeconomic status indicators.

	Sensitivity Analysis 1[a]	Sensitivity Analysis 2[b]	Sensitivity Analysis 3[c]
	Percent Increase (95 % CI)	Percent Increase (95 % CI)	Percent Increase (95 % CI)
Non-accidental			
PM_{10} (per 10 μg/m³)	4.68 (3.62, 5.75)	5.22 (4.09, 6.36)	5.69 (4.53, 6.86)
NDVI (per IQR)[d]	0.44 (-0.78, 1.67)	-0.07 (-1.35, 1.22)	-0.96 (-2.26, 0.35)
Cardiovascular disease			
PM_{10} (per 10 μg/m³)	9.59 (7.57, 11.65)	10.28 (8.22, 12.38)	10.79 (8.64, 13.00)
NDVI (per IQR)	-1.51 (-3.64, 0.68)	-2.07 (-4.25, 0.15)	-2.84 (-5.05, -0.57)
Ischaemic heart disease			
PM_{10} (per 10 μg/m³)	7.62 (4.30, 11.04)	8.13 (4.77, 11.61)	9.36 (5.94, 12.91)
NDVI (per IQR)	-2.36 (-5.84, 1.25)	-2.91 (-6.40, 0.71)	-4.13 (-7.61, -0.51)
Respiratory disease			
PM_{10} (per 10 μg/m³)	-2.90 (-5.14, -0.60)	-2.70 (-4.94, -0.40)	-1.83 (-4.07, 0.47)
NDVI (per IQR)	2.13 (-0.50, 4.83)	1.53 (-1.08, 4.21)	0.03 (-2.54, 2.68)
Chronic lower respiratory disease			
PM_{10} (per 10 μg/m³)	15.50 (10.97, 20.20)	16.00 (11.46, 20.73)	15.70 (11.20, 20.38)
NDVI (per IQR)	-2.76 (-7.38, 2.09)	-3.07 (-7.66, 1.74)	-1.98 (-6.65, 2.94)
Lung cancer			
PM_{10} (per 10 μg/m³)	3.01 (0.95, 5.10)	3.21 (1.16, 5.31)	3.44 (1.36, 5.57)
NDVI (per IQR)	1.69 (-0.64, 4.07)	1.53 (-0.79, 3.91)	1.24 (-1.12, 3.66)

[a] Model adjusted for household income instead of percentage of adults with low education.
[b] Model adjusted for local tax per capita instead of percentage of adults with low education.
[c] Model adjusted for percentage of those who receive social benefit instead of percentage of adults with low education.
[d] IQR for NDVI = 0.20.

Table S4. Percent changes in cause-specific mortality per 10 μg/m^3 increase in PM_{10} by the level of greenness using models with different socioeconomic status indicators.

	Non-accidental	Cardio Vascular Disease	Ischaemic Heart Disease	Respiratory Disease	Chronic Lower respiratory Disease	Lung Cancer
Sensitivity analysis 1[a]						
Greenness[d]						
High	6.78 (4.86, 8.73)	9.01 (5.45, 12.68)	4.22 (-2.25, 11.13)	-0.86 (-4.65, 3.08)	20.09 (12.09, 28.88)	5.37 (1.31, 9.59)
Medium	4.33 (2.59, 6.10)	9.39 (6.08, 12.81)	7.06 (1.89, 12.49)	-2.64 (-6.17, 1.01)	14.36 (6.99, 22.24)	1.50 (-1.58, 4.67)
Low	3.28 (1.19, 5.43)	10.36 (6.48, 14.39)	7.86 (1.38, 14.76)	-8.99 (-12.69, -5.12)	11.06 (1.83, 21.13)	4.76 (0.48, 9.23)
Sensitivity analysis 2[b]						
Greenness[d]						
High	6.50 (4.60, 8.44)	8.37 (3.98, 12.95)	4.82 (-1.72, 11.80)	-1.00 (-4.89, 3.04)	20.07 (11.94, 28.79)	4.95 (0.98, 9.08)
Medium	4.15 (2.43, 5.89)	9.17 (5.96, 12.47)	6.02 (1.08, 11.19)	-3.73 (-7.49, 0.18)	13.51 (6.28, 21.24)	1.20 (-1.89, 4.40)
Low	5.60 (3.27, 7.98)	15.87 (11.38, 20.54)	12.64 (5.77, 19.94)	-8.55 (-12.30, -4.65)	15.06 (5.47, 25.52)	5.76 (1.50, 10.21)
Sensitivity analysis 3[c]						
Greenness[d]						
High	8.20 (6.16, 10.27)	10.36 (6.77, 14.07)	5.95 (-0.74, 13.08)	0.65 (-3.23, 4.69)	20.21 (12.39, 28.58)	5.40 (1.33, 9.63)
Medium	4.90 (3.10, 6.74)	9.79 (6.43, 13.26)	7.82 (2.62, 13.29)	-2.36 (-5.89, 1.30)	14.33 (6.95, 22.20)	1.70 (-1.40, 4.90)
Low	5.78 (3.53, 8.08)	15.53 (11.22, 20.03)	12.46 (5.81, 19.51)	-7.24 (-10.99, -3.34)	16.37 (6.85, 26.72)	5.68 (1.55, 9.97)

[a] Model adjusted for household income instead of percentage of adults with low education.

[b] Model adjusted for local tax per capita instead of percentage of adults with low education.

[c] Model adjusted for percentage of those who receive social benefit instead of percentage of adults with low education.

[d] Greenness was based on the NDVI value at each district level. A high group was defined as those with values ≥ 66th percentile, medium group as those with values ≥ 33th percentile, low group as those with values < 33th percentile.

Table S5. Percent changes in cause-specific mortality and 95 % confidence interval for IQR increase in NO_2 and SO_2 in single- and two-pollutant model.

	Non-Accidental	Cardio Vascular Disease	Ischaemic Heart Disease	Respiratory Disease	Chronic Lower Respiratory Disease	Lung Cancer
Single pollutant (NO_2)[a]	3.55 (1.82, 5.32)	8.09 (4.76, 11.53)	6.18 (1.15, 11.46)	-5.09 (-8.42, -1.63)	12.56 (5.27, 20.36)	1.56 (-1.54, 4.76)
+ PM_{10}	1.81 (0.13, 3.53)	4.20 (1.09, 7.41)	3.29 (-1.69, 8.53)	-4.00 (-7.52, -0.34)	5.63 (-1.18, 12.91)	0.41 (-2.75, 3.66)
Single pollutant (SO_2)[b]	1.12 (0.01, 2.23)	1.10 (-0.95, 3.20)	0.57 (-2.52, 3.77)	-0.47 (-2.73, 1.84)	6.10 (1.65, 10.75)	2.16 (0.14, 4.22)
+ PM_{10}	0.98 (-0.04, 2.02)	0.80 (-1.08, 2.72)	0.47 (-2.63, 3.67)	-0.38 (-2.63, 1.93)	5.01 (0.92, 9.26)	2.25 (0.22, 4.32)

[a] IQR for NO_2 = 10.35

[b] IQR for SO_2 = 2.10

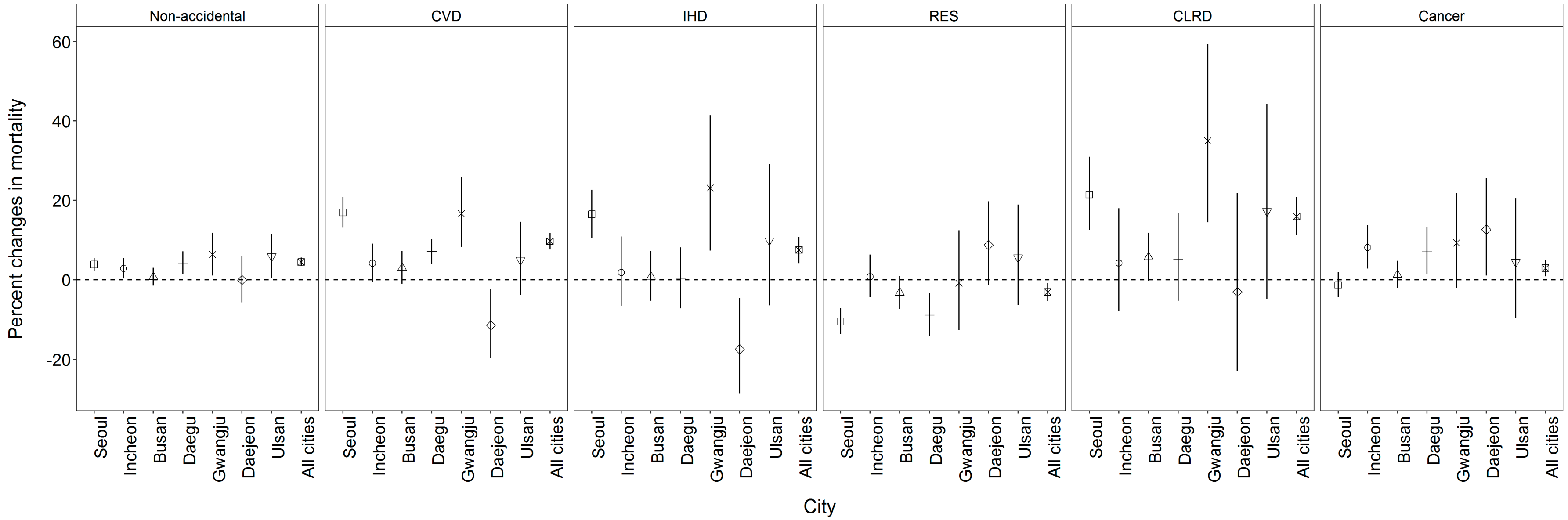

Figure S18. City-specific percent changes in cause-specific mortality and 95 % confidence interval for 10 μg/m^3 increase in PM_{10}.

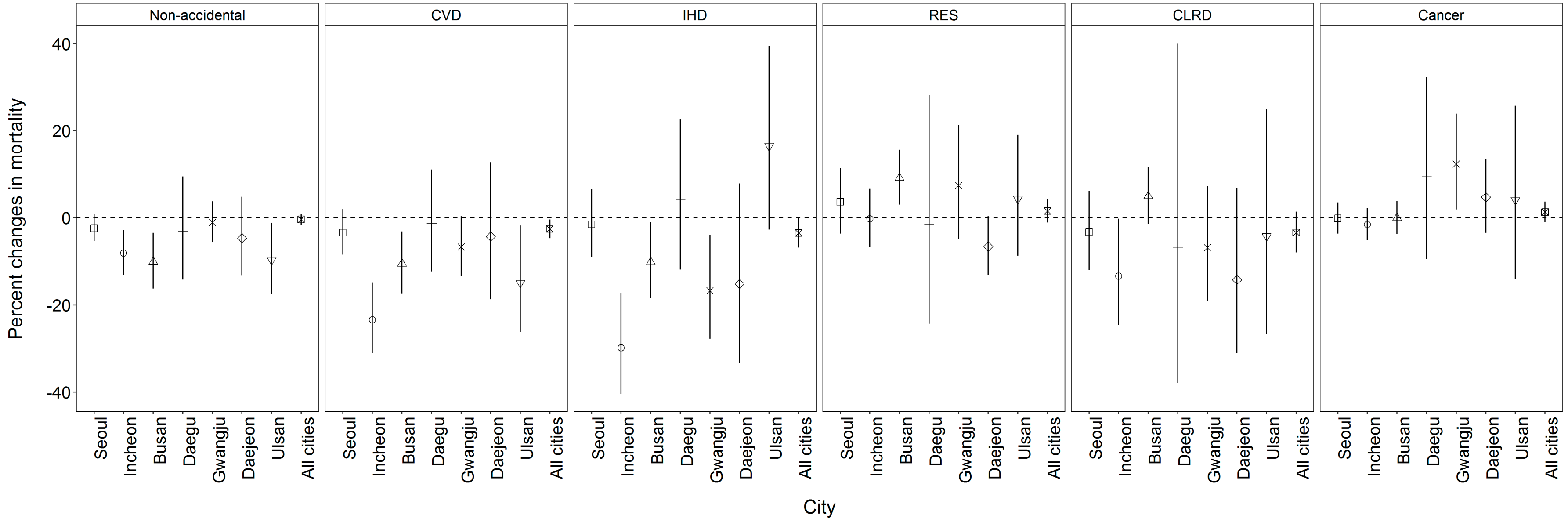

Figure S19. City-specific percent changes in cause-specific mortality and 95 % confidence interval for IQR increase in NDVI.
